# Transmisssion Dynamics of *Enterococcus* spp. Throughout the *Heliconius erato phyllis* (Lepidoptera; Nymphalidae) Life Cycle

**DOI:** 10.1111/1758-2229.70336

**Published:** 2026-04-07

**Authors:** Rosana Huff, Janira Prichula, Luana Silva Dornelles, Rebeca Inhoque Pereira, Jeverson Frazzon, Ana Paula Guedes Frazzon

**Affiliations:** ^1^ Programa de Pós‐Graduação em Microbiologia Agrícola e Do Ambiente, Instituto de Ciências Básicas da Saúde Universidade Federal do Rio Grande do Sul Porto Alegre Brazil; ^2^ Department of Ophthalmology, Mass Eye and Ear Harvard Medical School Boston Massachusetts USA; ^3^ Department of Microbiology Harvard Medical School Boston Massachusetts USA; ^4^ Departamento de Microbiologia, Imunologia e Parasitologia, Instituto de Ciências Básicas da Saúde Universidade Federal do Rio Grande do Sul Porto Alegre Brazil; ^5^ Departamento de Microbiologia, Instituto de Ciência e Tecnologia de Alimentos Universidade Federal Do Rio Grande Do Sul Porto Alegre Brazil

**Keywords:** antimicrobial resistance, butterfly, enterococci, horizontal transmission, insect gut microbiota

## Abstract

*Enterococcus* spp. are members of the gut microbiota of *Heliconius* butterflies, although their transmission remains poorly characterised*.* We used pulsed‐field gel electrophoresis (PFGE) to compare *Enterococcus* strains from developmental stages of *Heliconius erato phyllis* and larval diet leaves, assessing their presence across the life cycle. Two female butterflies were collected, and 432 enterococci were isolated from eggs, larval instars, pupae, adults and larval diet leaves. Species were identified by MALDI‐TOF MS, and antimicrobial susceptibility profiles were determined. *Enterococcus faecalis* and *Enterococcus casseliflavus* predominated across the butterfly life cycle and larval diet leaves, with isolates showing diverse resistance profiles. To minimise redundancy, isolates from the same sample, belonging to the same species and showing identical antimicrobial resistance profiles were considered closely related; therefore, 52 of 432 enterococci (24 *E. casseliflavus* and 28 *E. faecalis* ) were selected for genetic characterisation. PFGE revealed genetic relatedness between leaf‐ and larva‐derived strains, consistent with diet‐mediated horizontal transmission. In addition, related profiles between isolates from developmental stages may suggest persistence of enterococci across the life cycle. These findings demonstrate the prevalence of *E. faecalis* and *E. casseliflavus* throughout the life cycle of *H. erato phyllis* and suggest that diet‐associated horizontal transmission may contribute to their maintenance.

## Graphical abstract

*Enterococcus* spp. dominate the gut microbiota of *Heliconius erato phyllis*, transmitted horizontally via host plants ( *Passiflora suberosa* ) and persisting through development. Multidrug‐resistant strains were found in larvae and plants, suggesting environmental reservoirs of antimicrobial resistance. This study reveals key microbial transmission dynamics and adaptation in butterflies.

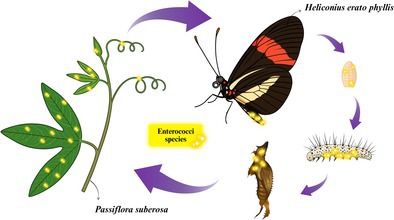

## 1 Introduction

The butterfly *Heliconius erato phyllis* (Lepidoptera; Nymphalidae) is a model organism in genetic and evolutionary studies and is widely distributed in South America (Mattila et al. 2021). This species undergoes a holometabolous life cycle (complete metamorphosis) comprising four distinct stages: egg, larva, pupa and adult. The larval stage represents the primary feeding and growth phase and is subdivided into five distinct instars (L1–L5) (van Belleghem et al. 2021). Adult females are monandric, mating only once and lay eggs individually on the apical meristems of host plants in the family Passifloraceae. Larvae in the L1–L5 instars primarily feed on leaves of *Passiflora misera, Passiflora suberosa* and *Passiflora capsularis* (Rodrigues and Moreira 2002), which are native plants known to contain cyanogenic glycosides. These toxic compounds are assimilated by the larvae, rendering them unpalatable to predators (de Castro et al. 2019, 2020). Additionally, *H. erato phyllis* adults are able to *de novo* biosynthesise cyanogenic glycosides, which are subsequently transferred to their eggs (Hay‐Roe and Nation 2007; Mattila et al. 2021). Beyond these well‐characterised chemical defences, increasing evidence suggests that host‐associated microorganisms may also influence physiological processes across insect development (Hu et al. 2022; Zhang et al. 2022; Shao et al. 2024).

Microbiota studies within Lepidoptera, particularly in *Heliconius* spp., consistently report a predominance of Firmicutes, with *Enterococcus* spp. constituting the dominant taxon (Vilanova et al. 2016; van Schooten et al. 2018; Hammer et al. 2020; Luna et al. 2023; Huff et al. 2024). *Enterococcus* species are Gram‐positive, catalase‐negative cocci widely distributed across diverse habitats, including soil, water and plants, and are commonly found in the gastrointestinal tracts of vertebrates and invertebrates. To date, over 90 species of *Enterococcus* have been described (LPSN 2026). Among them, *Enterococcus faecalis, Enterococcus casseliflavus, *Enterococcus* flavescens, Enterococcus mundtii* and *Enterococcus gallinarum* are frequently isolated from insects (Hammer et al. 2014; Vilanova et al. 2016; Allonsius et al. 2019; LPSN 2026; Huff et al. 2020). Recent taxonomic advances have described two new species phylogenetically proximate to *E. casseliflavus*: *Enterococcus innesii*, isolated from the gut of *Galleria mellonella* (Gooch et al. 2021) and *Enterococcus entomosocium*, recovered from *Spodoptera frugiperda* larvae which has the potential to degrade xenobiotic pesticides (Gomes et al. 2023). Within Lepidoptera, *E. casseliflavus* is recurrently encountered as a dominant gut symbiont in specialist herbivores such as *Hyles euphorbiae* and *Brithys crini*, whose diets are enriched in latex‐ and alkaloid‐bearing host plants, respectively (Vilanova et al. 2016).

Despite the frequent occurrence and functional relevance of enterococci in insect hosts, their origin and transmission dynamics remain poorly understood (Paniagua Voirol et al. 2018; Huff et al. 2020). Insect microbiota may be acquired through multiple routes, such as host behaviour and ecology (Moran et al. 2019). In Lepidoptera, horizontal transmission, via ingestion of contaminated food, environmental exposure or social interaction, is considered the predominant mode of microbiota acquisition (Strano et al. 2018). However, vertical transmission from parent to offspring, particularly via egg surfaces, has also been documented in certain species (Schmidt and Engel 2021). While vertical transmission of *Enterococcus* spp. has been demonstrated in some Lepidoptera through oviposition, this mechanism has yet to be confirmed in *Heliconius* butterflies (Brinkmann et al. 2008; Chen et al. 2016). As a result, the persistence of these bacteria throughout the developmental stages of *H. erato phyllis* remains an open question.

In a previous study by our group, clonal relationships among *Enterococcus* isolates were observed in the faeces of L5 sibling larvae of *H. erato phyllis*; however, the route of bacteria acquisition, horizontal versus vertical, could not be determined (Huff et al. 2020). To address this knowledge gap, the present study was designed to investigate the route of transmission of *Enterococcus* spp. in the *H. erato phyllis* life cycle. Specifically, we aimed to answer the following question: Are *Enterococcus* spp. transmitted in the life cycle of *H. erato phyllis* through vertical transmission, horizontal acquisition or a combination of both mechanisms?

## 2 Materials and Methods

### 2.1 Butterflies' Collection

Fertilised females of *H. erato phyllis* (*n* = 2) were captured from a wild population in two Atlantic Forest fragments located in Rio Grande do Sul, southern Brazil: parental generation 1 (PG1) (Porto Alegre; 30°04′29.8″S, 51°06′47.8″W) and PG2 (Aguas Belas; 30°02′21.4″S, 51°01′29.7″W), during January and February 2020.

Following capture, the butterflies were individually housed under semi‐natural conditions in insectaries at the Department of Genetics, Universidade Federal do Rio Grande do Sul, Porto Alegre, RS, Brazil. Each insectary was provided with the main host plants, including *P. suberosa* , which served as an oviposition substrate. Adults were fed daily with a nutrient mixture consisting of commercial honey bee pollen and honey diluted in water at a 2:1:7 ratio.

This study was carried out according to the recommendations of the Chico Mendes Institute for Biodiversity Conservation (ICMBio). The protocol was approved by the Information Authorisation System in Biodiversity (SISBIO) number 68730‐2.

### 2.2 Butterflies' Life Cycle Sample Collection

**FIGURE 1:**
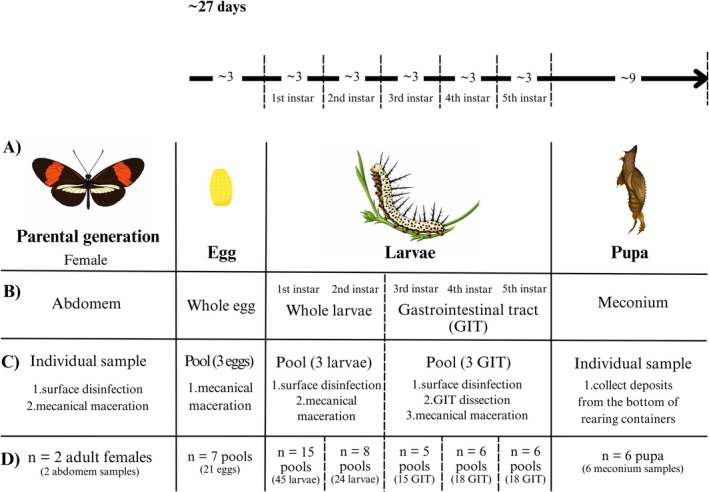
Schematic representation of the sampling methodology across the life cycle stages of *Heliconius erato phyllis*. (A) Life cycle stages (parental generation, egg, larvae [first to five instars], pupa and newly emerged adult); (B) Type of biological sample collected; (C) Sampling methodology and (D) Sample size and composition for each life stage.

The life cycle of *H. erato phyllis* was monitored daily and samples were collected from the following developmental stages: eggs (E), five larval instars (L1–L5), pupa (P) and PG (Figure 1A). The average life cycle duration was 27 ± 3 days, comprising an incubation period of approximately 3 days, a larval phase lasting 15 ± 1 days, and a pupa phase during 9 ± 2 days. During the larval stages, individuals were fed leaf fragments of *P. suberosa* .

**FIGURE 2:**
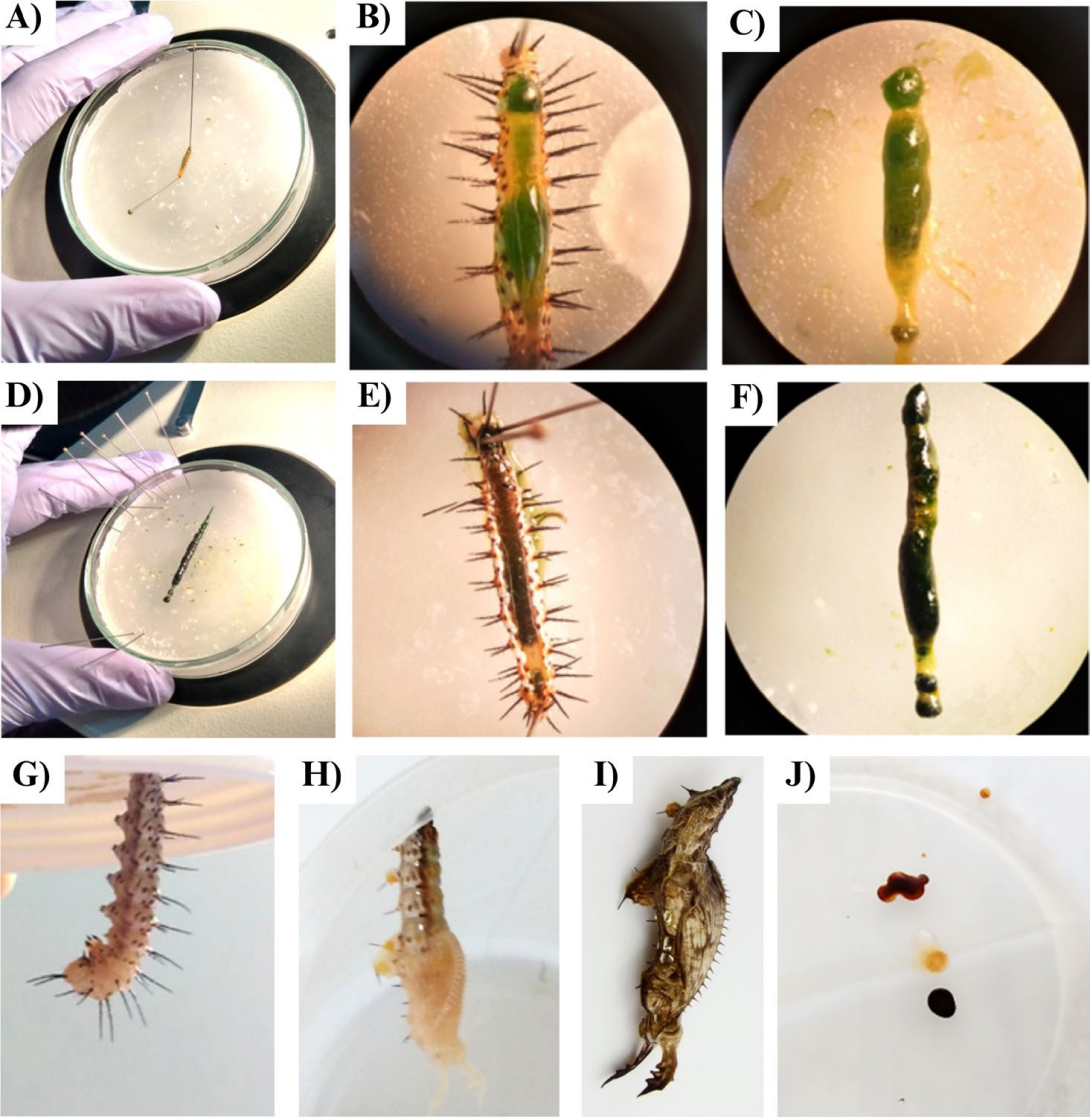
Sampling methodology of *Heliconius erato phyllis*. (A) L3 instar immobilised in a Petri dish before dissection; (B) L3 instar with dorsal cuticle incision exposing the intact gut; (C) L3 instar gut tract (foregut, midgut and hindgut). (D) L4 instar gut tract (foregut, midgut and hindgut); (E) L5 instar with dorsal cuticle incision, revealing digestive system; (F) L5 instar gastrointestinal tract (foregut, midgut and hindgut); (G) L5 instar in prepupal‐stage exhibiting characteristic morphological changes; (H) Newly formed pupa (< 1 h postpupation); (I) Mature pupa and (J) Meconium deposits following adult eclosion.

Samples from eggs and L1 and L2 instars consisted of pools of three individuals each (Figure 1B–D). To assess potential differences in microbial acquisition, L1 instars were divided into two experimental groups: those fed on host plant material (L1‐F) and those fed exclusively on egg chorion posteclosion (L1‐C). Eggs were collected using a paintbrush, disinfected with 70% ethanol and subsequently macerated. L1 and L2 instars underwent surface disinfection by sequential immersion in 1% sodium hypochlorite (1 min), sterile distilled water (1 min), 70% ethanol (1 min), followed by a final rinse in sterile distilled water (1 min). For L3, L4 and L5 instars, samples consisted of pools of dissected gastrointestinal tracts, including foregut, midgut and hindgut, each pool composed of three individuals (Figures 1B–D, 2A–F). Before dissection, larvae were anaesthetised at −20°C for 5 min. Additionally, individual faecal samples were collected from both L4 and L5 instars.

Samples from the pupal stages and PG consisted of one individual (Figure 1B–C). Pupal samples (P) were represented by meconium deposits collected from the bottom of rearing containers immediately following adult emergence (Figure 2G–J). PG samples consisted of the entire abdomen. Before sampling, adults were anaesthetised and subjected to surface disinfection following the same protocol applied to earlier instars.

The total number of samples collected is summarised in Table S1.

### 2.3 Isolation and Identification of *Enterococcus* Species

The isolation of enterococci was performed as described by Santestevan et al. (2015), with modifications. All samples were macerated using a sterile pestle in 1.5 mL microtubes containing 400 μL of peptone water. Briefly, 100 μL of each sample was inoculated onto m‐Enterococcus Agar Base (Himedia, Mumbai, India) and incubated aerobically at 37°C for 24 h. Colonies showing purple/red colour in m‐Enterococcus Agar Base plates were selected and inoculated into Brain Heart Infusion (BHI) broth, followed by incubation at 37°C for 48 h. Isolated colonies that tested negative for catalase activity and exhibited Gram‐positive diplococcal morphology were stored at −20°C in a suspension containing 10% (w/v) skim milk powder (Molico; Difco, Sparks, MD, USA) and 10% (v/v) glycerol (Neon Comercial Ltda., São Paulo, SP, Brazil).

Species‐level identification was performed by matrix‐assisted laser desorption/ionisation time‐of‐flight mass spectrometry (MALDI‐TOF MS) using a Microflex LT mass spectrometer (Bruker Daltonik GmbH, Germany). MALDI‐TOF MS allows rapid and accurate bacterial identification based on species‐specific protein mass spectra, predominantly derived from ribosomal proteins, and has been shown to reliably discriminate between *Enterococcus* species. Spectra were acquired and analyzed using Bruker BioTyper 1.1 software and matched against the manufacturer's reference database. Identification scores were interpreted according to the manufacturer's criteria, with values ≥ 2.0 indicating reliable species‐level identification (Sauget et al. 2017).

### 2.4 Antimicrobial Susceptibility Profiles of *Enterococcus* Strains

The antimicrobial susceptibility of enterococcal strains isolated from *H. erato phyllis* and leaves was determined by the Kirby‐Bauer disk diffusion method on Mueller–Hinton agar, following the Clinical and Laboratory Standards Institute guidelines (CLSI [Clinical and Laboratory Standards Institute] 2021). Twelve antibiotics representing eight classes were evaluated: ampicillin (10 μg; AMP), vancomycin (30 μg; VAN), erythromycin (15 μg; ERY), tetracycline (30 μg; TET), ciprofloxacin (5 μg; CIP), norfloxacin (10 μg; NOR), nitrofurantoin (300 μg; NIT), chloramphenicol (30 μg; CHL), high‐level gentamicin (120 μg; GEN), linezolid (30 μg; LNZ), rifampicin (5 μg; RIF) and high‐level streptomycin (300 μg; STR). *E. faecalis* ATCC 29212 served as the quality control strain.

Isolates displaying intermediate and resistant phenotypes were collectively classified as resistant. Resistance patterns were categorised as: single‐drug resistant (SR, resistant to one antimicrobial class), double‐drug resistant (DR, resistant to two antimicrobial classes) or multidrug‐resistant (MDR, resistant to three or more antimicrobial classes) according to Magiorakos et al. (2012).

### 2.5 Molecular Typing of Enterococci by Pulsed‐Field Gel Electrophoresis (PFGE)

*Enterococcus* strains isolated from the *H. erato* *phyllis* life cycle and from leaves were selected for genetic relationship analysis using PFGE. Strain selection for PFGE analysis was based on five criteria: (i) *Enterococcus* species, (ii) antimicrobial resistance profile, (iii) maternal origin (PG1 or PG2), (iv) developmental stage of *H. erato phyllis* and (v) origin from larval diet leaves. These criteria were applied to reflect the observed diversity while minimising clonal redundancy, considering isolates of the same species from the same environment and exhibiting identical antimicrobial resistance profiles as closely related.

Chromosomal DNA from enterococci was extracted and electrophoresed according to the protocols described by Murray et al. (1990) and Saeedi et al. (2002), using the restriction enzyme *SmaI* (Invitrogen). Electrophoresis was performed using a CHEF‐DRII system (Bio‐Rad Laboratories, Richmond, CA, USA) with the following parameters: ramped pulse times recommended by Saeedi et al. (2002) and constant temperature at 11°C.

### 2.6 Cluster Analysis

Gel images and DNA band profiles were analysed using the GelCompar II software system (version 6.6; Applied Maths, Sint‐Martens‐Latem, Belgium) (optimisation 1.0, tolerance 1.0, cut off %90). DNA banding patterns were normalised using lambda concatemer ladder standards. Dice correlation coefficient was used to calculate similarity for band analysis and Unweighthed Pairwise Grouping Mathematical Averaging (UPGMA) was used for cluster analysis. A dendrogram was constructed to assess genetic relatedness among isolates, applying an 80% similarity cutoff, as proposed by Tenover et al. (1995).

## 3 Results

In this study, we investigated the transmission routes of *Enterococcus* throughout the life cycle of *H. erato phyllis*. To assess this transmission, we analysed the distribution of *Enterococcus* species and their antimicrobial susceptibility profiles across all developmental stages, as well as in the leaves used to feed the immature forms. Based on this information, representative isolates were selected for PFGE, and their clonal relation.

### 3.1 Distribution of Enterococci Species in *H. erato Phyllis* Life Cycle and Passion Flowers Leaves

*Enterococcus* spp. were isolated from 54 (72%) of the 75 samples analysed in this study (Table S2). A total of 351 enterococci were isolated from samples collected across all life stages of the butterfly life cycle, and *E. faecalis* (*n* = 161; 44.28%) and *E. casseliflavus* (*n* = 147; 43.1%) were the predominant species in the samples. The remaining isolates included *E. mundtii* (*n* = 22; 6.45%), *Enterococcus hirae* (*n* = 20; 5.86%) and *Enterococcus faecium* (*n* = 1; 0.3%), all of which were found at considerably lower frequencies.

*Enterococcus* strains were recovered in the life cycle of *H. erato phyllis*, with distinct distributions observed across developmental stages (Table 1). *E. faecalis* was the unique species found in the PG (*n* = 37; 10.54%) and L1‐C instars (*n* = 16; 4.55%). *E. hirae* was only detected in E and L1‐F instars. On the other hand, *E. casseliflavus* was the main species in L1‐F (*n* = 28; 7.94%) and L2 instars (*n* = 38; 10.83%), as well as in the P stage (*n* = 45; 12.82%). In L3 instars, *E. casseliflavus* (*n* = 10; 2.84%) and *E. mundtii* (*n* = 12; 3.41%) were detected at similar frequencies. During the L4 and L5 instars, *E. faecalis* became the dominant species, with 48 isolates (13.67%) and 60 isolates (17.09%) recovered, respectively (Table 1).

**TABLE 1:** Distribution of *Enterococcus* species in the life cycle of *Heliconus erato phyllis* and from passion flower leaves used to feed the immature forms.

Species	Life cycle of *Heliconus erato phyllis*									Leaf	Total
	PG	E	L1‐C	L1‐F	L2	L3	L4	L5	P		
*Enterococcus casseliflavus*	0	0	0	28	38	10	16	10	45	49	196
*Enterococcus faecalis*	37	0	16	0	0	0	48	60	0	32	193
*Enterococcus mundtii*	0	0	0	0	0	12	0	10	0	0	22
*Enterococcus hirae*	0	10	0	10	0	0	0	0	0	0	20
*Enterococcus faecium*	0	1	0	0	0	0	0	0	0	0	1
Total	37	11	16	38	38	22	64	80	45	81	432

Abbreviations: E, eggs; L1‐C, first instar fed exclusively on egg chorion posteclosion; L1‐F, first instar fed with host plant material; L2–L5, larval instars; P, pupa—meconium; PG, parental generations.

From the 19 *P. suberosa* leaf samples, a total of 81 enterococci were obtained. *E. casseliflavus* was the most prevalent species (*n* = 49; 60.5%), followed by *E. faecalis* (*n* = 32; 39.5%) (Table 1).

The complete data on the samples collected in the study and the species of *Enterococcus* isolated can be found in Tables S2–S4.

### 3.2 Antimicrobial Susceptibility Profiles of Enterococci Isolated From *H. erato Phyllis* Life Cycle and Passion Flower Leaves

Of the 432 *Enterococcus* isolates obtained in this study, 339 (78.5%) exhibited resistance to at least one of the antimicrobials tested. The distribution of antimicrobial susceptibility across species is shown in Table 2.

**TABLE 2:** Antibiotic susceptibility profiles of *Enterococcus* species isolated from life cycle of *Heliconus erato* phyllis, and from the passion flower leaves used to feed the larvae.

Enterococcus species (*n*)	Number of resistant strains to^a^									Profile^b^		
	ERY	QUI	RIF	LZD	STR	GEN	NIT	TET	CHL	SR	DR	MDR
*H. erato phyllis*												
*Enterococcus casseliflavus* (147)	48	31	38	2	2	0	0	0	0	52	27	7
*Enterococcus faecalis* (161)	133	98	103	3	0	1	0	0	1	12	57	81
*Enterococcus mundtii* (22)	10	1	0	0	0	0	0	0	0	11	0	0
*Enterococcus hirae* (20)	3	3	1	4	0	0	2	0	0	5	2	1
*Enterococcus faecium* (1)	1	0	0	0	0	0	0	0	0	1	0	0
Subtotal (351)	195	133	142	9	2	1	2	0	1	81	86	89
Passion flower leaves												
*E. casseliflavus* (49)	22	20	15	0	0	0	0	1	0	16	3	14
*E. faecalis* (32)	21	7	29	5	0	0	0	0	0	9	12	9
Subtotal (81)	43	27	44	5	0	0	0	1	0	25	15	23
Total (432)	238	160	186	14	2	1	2	1	1	106	101	112

^a^ Antibiotics: CHL, chloramphenicol; ERY, erythromycin; GEN, gentamicin; LZD, linezolid; NIT, nitrofurantoin; QUI, ciprofloxacin and/or norfloxacin; RIF, rifampicin; STR, streptomycin; TET, tetracycline.

^b^ Resistance profiles: DR, double resistance; MDR, multidrug resistance; SR, single resistance.

Among the 351 strains isolated throughout the butterfly's life cycle, resistance to erythromycin (55.5%; *n* = 195/351) was the most frequent, followed by rifampicin (40.4%; *n* = 142/351) and quinolones (37.9%; *n* = 133/351). Lower resistance rates were observed for linezolid (2.6%), streptomycin (0.5%), nitrofurantoin (0.5%), gentamicin (0.2%) and chloramphenicol (0.2%). Notably, all isolates were susceptible to tetracycline, vancomycin and ampicillin. Regarding resistance profiles, single resistance (SR), double resistance (DR) and multidrug resistance (MDR) were detected in 23.1%, 24.5% and 25.4% of the isolates, respectively.

Throughout the butterflies' developmental stages, *Enterococcus* strains isolated from eggs exhibited a low frequency (27.2%) of antimicrobial resistance. In contrast, resistance was notably more prevalent among isolates from larvae and pupae, with over 50% of the strains showing resistance to at least one antimicrobial, particularly rifampicin and erythromycin. Strikingly, all isolates from the PG were resistant to rifampicin.

Additionally, among the 81 isolates obtained from *Passiflora* leaves, high resistance rates were observed for rifampicin (72.8%), erythromycin (65.4%) and quinolones (66.6%). In contrast, only one isolate (1.2%) showed resistance to chloramphenicol and linezolid, respectively.

### 3.3 Genetic Relationship Among Enterococci Isolates From the Life Cycles of *H. erato Phyllis*

Of the 432 *Enterococcus* isolates obtained in this study (351 from developmental stages and 81 from host plant leaves), 52 were selected for genetic characterisation by PFGE based on predefined criteria to minimise clonal redundancy. Among these, 24 were *E. casseliflavus* (12 from the butterfly life cycle and 12 from leaves) and 28 were *E. faecalis* (18 from the butterfly life cycle and 10 from leaves).

**FIGURE 3:**
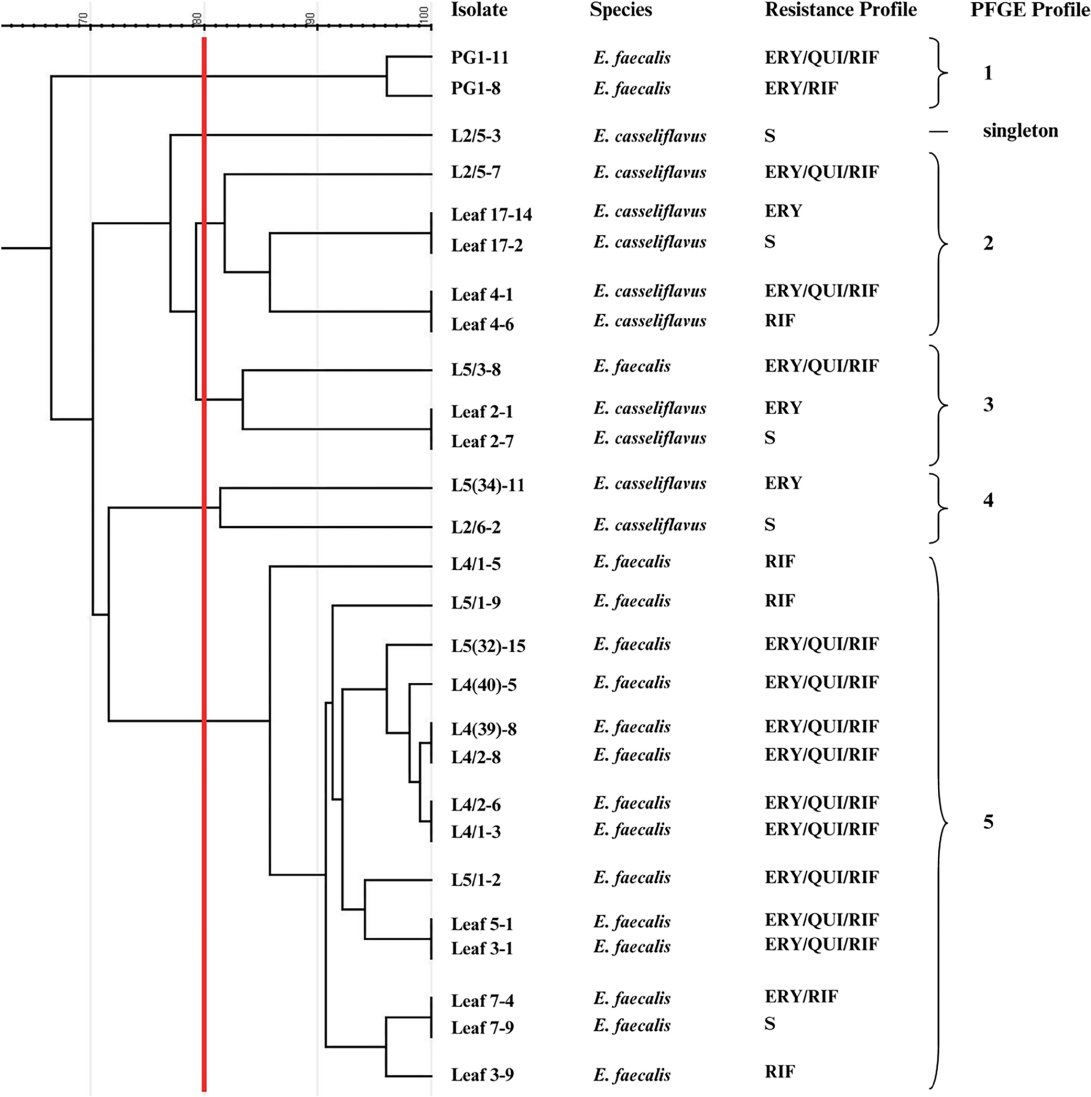
Cluster analysis dendrogram of *Heliconius erato phyllis* developmental stages and host plant leaves based on *Sma*I restriction fragment patterns obtained by pulsed‐field gel electrophoresis. Samples include progeny from parental generation 1 (PG1) and their corresponding larval food source leaves. Isolates/sample origin: PG1, parental generation 1; L2, 2nd instar larvae; L5, 5th instar larvae; L5(n), individual faecal sample from 5th instar larvae; L4, 4th instar larvae; L4 (n), individual faecal sample from 4th instar larvae. Antibiotics: ERY, erythromycin; QUI (ciprofloxacin and/or norfloxacin); RIF, rifampicin; S, susceptible. The cutoff at the 80% level of genetic similarity is indicated by a vertical red line.

Cluster analysis of strains isolated from the PG1 lineage using a similarity threshold of ≥ 80% revealed six distinct profiles (Figure 3). Among them, only one strain of *E. casseliflavus* susceptible to all antimicrobial tests isolated from an L2 instar was classified as a singleton. Profile 1 includes only two *E. faecalis* strains isolated from PG. These strains exhibited high genetic heterogeneity compared to *E. faecalis* isolates obtained from the second generation of the same parental lineage. Profiles 2, 3 and 5 grouped *E. casseliflavus* and *E. faecalis* strains from L2, L4 and L5 instars and *P. suberosa* leaf samples. It is interesting to note that, in profile 4, *E. casseliflavus* strains exhibiting genetic similarity were isolated from different larval instars (L2 and L4).

**FIGURE 4:**
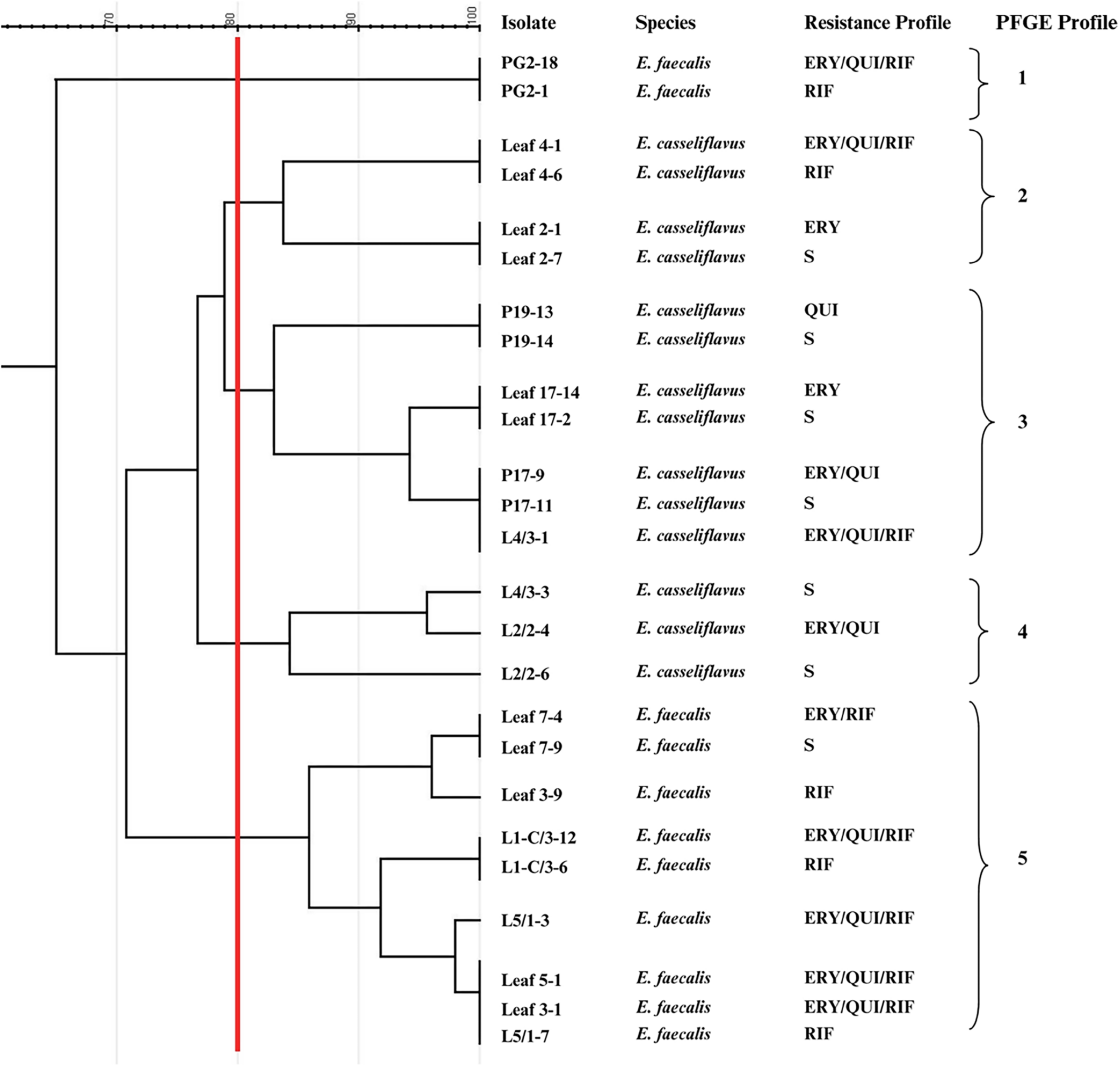
Cluster analysis dendrogram of *Heliconius erato phyllis* developmental stages and host plant leaves based on *Sma*I restriction fragment patterns obtained by pulsed‐field gel electrophoresis. Samples include progeny from parental generation 2 (PG2) and their corresponding larval food source leaves. Isolates/sample origin: PG2, parental generation 2; P, pupa/meconium; L4, 4th instar larvae; L1‐C, 1st instar larvae that ingested only chorion; L5, 5th instar larvae. Antibiotics: ERY, erythromycin; QUI (ciprofloxacin and/or norfloxacin); RIF, rifampicin; S, susceptible. The cutoff at the 80% level of genetic similarity is indicated by a vertical red line. The cutoff at the 80% level of genetic similarity is indicated by a vertical red line.

For all lineages from the PG2, five profiles were identified (Figure 4). As observed for PG1, profile 1 grouped *E. faecalis* strains from the adult female, genetically unrelated to other isolates. In profile 2, *E. casseliflavus* strains from different *P. suberosa* leaf samples clustered together. In profile 3, *E. casseliflavus* isolates with PFGE‐indistinguishable profiles were detected in both the pupa (sample P17) and L4 instars, suggesting possible persistence of closely related isolates across larval and postmetamorphic stages. In contrast, profile 4 aggregated strains were isolated from L2 and L4 instars. The PFGE profile 5 grouped *E. faecalis* strains from newly hatched larvae (L1‐C), which had only ingested egg chorion, as well as strains isolated from *P. suberosa* leaves and from L5 instars.

## 4 Discussion

The results of this study report the transmission dynamics of *Enterococcus* species across the developmental stages of *H. erato phyllis*. Our findings suggest that horizontal transmission likely contributes to the persistence and dissemination of enterococci species throughout the butterfly's life cycle. By documenting the occurrence, antimicrobial resistance profiles and clonal relationships of *Enterococcus* isolates across all developmental stages, this study provides evidence for the continuity of enterococci across host development. To our knowledge, this is the first study to assess the presence and transmission patterns of enterococci across the complete life cycle of *H. erato phyllis*, offering novel insights into their ecological dynamics without inferring functional effects on host fitness.

In the present study, *Enterococcus* strains were isolated from all life stages of *H. erato phyllis* butterflies, aligning with previous findings that report the presence of enterococci in Lepidoptera. One of the few studies focusing on the microbiota of nonagricultural pest Lepidoptera, by Hammer et al. (2014), identified *Enterococcus* as the most abundant genus throughout all developmental stages of *Heliconius* butterflies from Panama. Similarly, Teh et al. (2016) demonstrated in vivo the transmission route of *E. mundtii* in *Spodoptera littoralis*, detecting the bacterium at every life stage, including eggs and during metamorphosis. Additional studies have shown that *Enterococcus* spp. frequently dominate the gut microbiota of Lepidoptera larvae and may regulate gut microbial communities (Vilanova et al. 2016; Shao et al. 2017; Huff et al. 2024). Enterococci can tolerate toxic plant compounds such as alkaloids present in the insects' host plants (Vilanova et al. 2016). Chen et al. (2016) found that *Enterococcus* spp. stably colonise the larval gut of *S. littoralis* , contributing to nutrient acquisition, particularly terpenoid and polyketide metabolism and bolstering host defence through antimicrobial production by *E. mundtii* .

Enterococcal isolates recovered throughout the life cycle of *H. erato phyllis* exhibited phenotypic resistance to erythromycin, rifampicin and quinolones, with antimicrobial resistance varying across developmental stages. While isolates from eggs showed low frequency, those from larvae and pupae demonstrated a marked increase in resistance prevalence. The progressive increase in resistance throughout insect development may reflect shifts in the relative abundance of resistant enterococcal populations and/or the acquisition of resistant strains through horizontal transmission from the environment, particularly via the larval diet. Supporting this hypothesis, enterococci isolated from *Passiflora* leaves, the exclusive component of the larval diet, displayed resistance profiles comparable to those observed in larval and pupal isolates, suggesting that ingestion of plant‐associated microbiota may contribute to the acquisition of resistant enterococci. Antimicrobial resistance profiles provide insights into both the ecological and epidemiological relevance of bacterial populations and have been used as auxiliary markers to differentiate isolates recovered from the same environment. Enterococci recovered from the same environment and exhibiting identical susceptibility profiles are suggestive of potential clonal relatedness, supporting the interpretation that environmental conditions may contribute to the persistence and dissemination of specific enterococcal lineages (Baldassarri et al. 2005). In epidemiological investigations, identical antibiograms have been used to infer potential transmission or relatedness among bacterial isolates, although genomic approaches remain the reference standard (Coll et al. 2025). In the present study, antimicrobial susceptibility profiles of isolates obtained across the life cycle of *H. erato phyllis* and from associated leaves were used to guide isolate selection and to minimise redundancy.

PFGE, a gold standard method for evaluating genetic relatedness among *Enterococcus* isolates, was applied to a representative subset obtained from different developmental stages of *H. erato phyllis* and from larval diet leaves. This subset encompassed isolates from all developmental stages, both maternal lineages, different *Enterococcus* species, distinct antimicrobial resistance profiles and isolates recovered from *P. suberosa* leaves. PFGE analysis across developmental stages revealed heterogeneous profiles, with clustering patterns consistent with diet‐associated acquisition from leaves and possible persistence of enterococcal populations throughout development. In PG1 (Figure 3), the clustering of *E. casseliflavus* and *E. faecalis* isolates from multiple larval instars together with diet‐associated isolates suggests horizontal transmission via the larval diet. Similar patterns were observed in PG2 (Figure 4), particularly in profiles 3–5, which demonstrated close genetic relationships between isolates recovered from *Passiflora* leaves and those isolated from larvae at different developmental stages. Additional evidence suggestive of persistence was observed for profile 4 in PG1 and profile 3 in PG2, which included isolates exhibiting close genetic relatedness across successive developmental stages (L1 and L5, as well as L4 and pupa). This observation is consistent with previous reports in other *Heliconius* species, in which enterococci detected in the larval gut were also recovered from meconium (Hammer et al. 2014).

Our findings showed that diet is the primary route of acquisition of enterococci in *H. erato phyllis*, consistent with patterns observed in other insects. Unlike vertebrates, where vertical transmission predominates, lepidopterans primarily acquire their microbiome through horizontal transmission from environmental exposure or diet. The persistent colonisation by enterococci across all life stages suggests these microbes possess unique adaptations to both the alkaloid‐rich diet and alkaline midgut environment characteristic of Lepidoptera. This ecological pattern is consistent with previous reports describing the prevalence of enterococci in related species (Vilanova et al. 2016; Huff et al. 2020), supporting their association with the lepidopteran gut ecosystem.

Although the data do not show the occurrence of vertical transmission of enterococci in *H. erato phyllis*, studies have documented this phenomenon in other lepidopteran species. Several studies have demonstrated that caterpillars can acquire bacterial symbionts by ingesting egg chorion material while emerging into the external environment. Brinkmann et al. (2008) reported that *Enterococcus* found in the gut of *Manduca sexta* larvae was acquired through ingestion of the chorion. Similarly, Chen et al. (2016) found that enterococci associated with adult *S. littoralis* females were also present on egg surfaces and later colonised the gut of larvae. Wang et al. (2020) further demonstrated that bacteria could be acquired from the chorion during hatching in *Grapholita molesta*. Although our findings do not support vertical transmission of enterococci in *H. erato phyllis*, we cannot rule out the possibility that other bacterial taxa and enterococcal species that remained uncultured under the experimental conditions used here may be vertically transmitted. The absence of postoviposition maternal care, along with the lack of specialised symbiont‐housing organs or social transmission mechanisms such as trophallaxis, likely constrains the potential for vertical transmission in this species. However, metagenomic approaches may be valuable for detecting bacterial taxa that could be involved in vertical transmission pathways.

In contrast, *E. faecalis* strains isolated from both PGs (profile 1 in PG1 and PG2) were genetically distinct from all other isolates, which may be compatible with acquisition from alternative environmental sources unrelated to the larval diet. Adult *Heliconius* butterflies undergo a marked dietary shift from leaf‐feeding larvae to pollen consumption, a rare trait among Lepidoptera. This adaptation is associated with increased longevity and fecundity and an expanded ecological niche (Gilbert 1972; Young and Montgomery 2020). Such a dietary shift could potentially introduce novel microbiota, including enterococci, which may influence the composition of adult gut microbial communities.

A limitation of this study is the use of a culture‐dependent approach combined with MALDI‐TOF MS identification, which may favour the recovery of dominant and readily cultivable *Enterococcus* species. Consequently, taxa present at low abundance, particularly during early developmental stages, may be underrepresented and occasional misidentification among closely related species cannot be entirely excluded. Another limitation concerns the use of PFGE to assess genetic relatedness, given that whole‐genome sequencing now provides higher resolution. Nevertheless, PFGE remains widely recognised as a gold‐standard method for evaluating genetic relatedness among *Enterococcus* isolates and offers several practical advantages over whole‐genome sequencing. It is rapid, reproducible and cost‐effective, allowing the simultaneous comparison of a large number of isolates with minimal analytical complexity. These features make PFGE particularly suitable for studies of population structure, clonal relationships and the identification of transmission patterns, providing meaningful insights even without the higher resolution of genomic sequencing (Neoh et al. 2019). Therefore, conclusions drawn from PFGE in this study should be interpreted as reflecting genetic relatedness within the resolution of the method, rather than exact sequence‐level equivalence.

## 5 Conclusions

Our study provides the first evidence that culturable *Enterococcus* spp., particularly *E. casseliflavus* and *E. faecalis* , are consistently associated with *H. erato phyllis* life cycle, including eggs, larval instars, pupa and adult females. The overlap of species composition and antimicrobial resistance profiles between strains isolated from larval instars and leaf suggests a key role of horizontal transmission, likely through diet. Collectively, our results suggest that *Enterococcus* spp. associated with *H. erato phyllis* may be acquired through horizontal transmission, particularly via the larval diet, with possible persistence across host developmental stages.

## Author Contributions

**Rosana Huff:** writing original draft, methodology, investigation, formal analysis, data curation. **Luana Silva Dornelles:** methodology, investigation. **Rebeca Inhoque Pereira:** methodology, investigation. **Janira Prichula:** formal analysis, original draft, writing. **Jeverson Frazzon:** writing, review and editing. **Ana Paula Guedes Frazzon:** data curation, project administration, editing, funding acquisition. All authors have read and agreed to the published version of the manuscript.

## Conflicts of Interest

The authors declare no conflicts of interest.

## Supporting information


**Table S1:** Samples collected during the experimental study of *Heliconius erato phyllis* life cycle.
**Table S2:** Samples of *Heliconius erato phyllis* collected and enterococci isolated in this study.
**Table S3:**. Distribution of *Enterococcus* species isolated according to sample origin (parental generation or leaf samples).
**Table S4:** Comparative distribution of *Enterococcus* species across life cycle stages of *Heliconius erato phyllis* populations (PG1 and PG2).

## Data Availability

The data that supports the findings of this study are available in the Supporting Information of this article.
