# Supplementary material for: Transmisssion Dynamics of Enterococcus spp. Throughout the Heliconius erato phyllis (Lepidoptera; Nymphalidae) Life Cycle
Source: Environ Microbiol Rep. 2026 Apr 7;18(2):e70336. doi: 10.1111/1758-2229.70336 (PMC13056423; doi:10.1111/1758-2229.70336)
Supplement: Supplementary file 1 — Table S1: Samples collected during the experimental study of Heliconius erato phyllis life cycle. Table S2: Samples of Heliconius erato phyllis collected and enterococci isolated in this study. Table S3:. Distribution of Enterococcus species isolated according to sample origin (parental generation or leaf samples). Table S4: Comparative distribution of Enterococcus species across life cycle stages of Heliconius erato phyllis populations (PG1 and PG2). [file EMI4-18-e70336-s001.docx]

**SUPPLEMENTARY MATERIALS**

**Supplementary Table 1**. Samples collected during the experimental study of *Heliconius erato phyllis* life cycle

|  |  |  | **Number of samples collected from** | |  |
| --- | --- | --- | --- | --- | --- |
| **Stage^1^** | **Sample Type** | **Sample** | **PG1** | **PG2** | **Total^2^** |
| PG | Individual | Abdominal homogenate | 1 | 1 | 2 |
| Egg | Pool | Whole-body homogenate | 5 | 2 | 7 |
| L1 | Pool | Whole-body homogenate | 11 | 4 | 15 |
| L2 | Pool | Whole-body homogenate | 6 | 2 | 8 |
| L3 | Pool | Dissected GIT^*^ | 4 | 1 | 5 |
| L4 | Pool | Dissected GIT^*^ | 3 | 3 | 6 |
|  |  | Fecal matter | 2 | 0 | 2 |
| L5 | Pool | Dissected GIT^*^ | 3 | 3 | 6 |
|  |  | Fecal matter | 1 | 3 | 4 |
| Pupa | Individual | Meconium | 3 | 6 | 9 |
| **Total** |  |  | **36** | **22** | **58** |

1: Life cycle abbreviations: PG (parental generation), PG1 (parental generation 1, Porto Alegre), PG2 (parental generation 2, Águas Belas). Developmental stages: L1-L5 (larval instars). 2: number of biological replicates. * GIT: gastrointestinal tract.

**Supplementary Table 2.** Samples of *Heliconius erato phyllis* collected and enterococci isolated in this study

| **Stage^1^** | **PG1** | | **PG2** | | **Total** | |
| --- | --- | --- | --- | --- | --- | --- |
|  | **collected** | **enterococci positive** | **collected** | **enterococci positive** | **collected** | **enterococci positive** |
| PG | 1 | 1 | 1 | 1 | 2 | 2 |
| E | 5 | 0 | 2 | 1 | 7 | 1 |
| L1-C | 6 | 0 | 1 | 1 | 7 | 1 |
| L1-F | 5 | 3 | 3 | 1 | 8 | 4 |
| L2 | 6 | 3 | 2 | 1 | 8 | 4 |
| L3 | 4 | 1 | 1 | 1 | 5 | 2 |
| L4 | 3 | 3 | 3 | 1 | 6 | 4 |
| L5 | 3 | 3 | 3 | 2 | 6 | 5 |
| P | 3 | 0 | 6 | 4 | 9 | 4 |
| Fecal | 5 | 27 | 11 | 0 | 17 | 27 |
| **Total** | **44** | **41** | **33** | **13** | **75** | **54** |

1. Life cycle abbreviations: PG1 (parental generation 1, Porto Alegre), PG2 (parental generation 2, Águas Belas). Life stage abbreviations: PG (parental generation), E (eggs), L1-C (1st instar fed exclusively on egg chorion post-eclosion), L1-F (1st instar fed with host plant material), L2-L5 (larval instars), P (pupa - meconium).

**Supplementary Table 3**. Distribution of *Enterococcus* species isolated according to sample origin (parental generation or leaf samples).

|  | **Number of enterococci** | | | | |
| --- | --- | --- | --- | --- | --- |
| **Sample origin (n)** | ***E. casseliflavus*** | ***E. faecalis*** | ***E. mundtii*** | ***E. hirae*** | ***E. faecium*** |
| *H. erato phyllis* |  |  |  |  |  |
| PG1^1^ (199) | 62 | 115 | 12 | 10 | 0 |
| PG2^1^ (152) | 85 | 46 | 10 | 10 | 1 |
| **Subtotal** | **147** | **161** | **22** | **20** | **1** |
| Leaves (81) | 49 | 32 | 0 | 0 | 0 |
| **Total (432)** | **196** | **193** | **22** | **20** | **1** |

1. Life cycle abbreviations: PG1 (parental generation 1, Porto Alegre), PG2 (parental generation 2, Águas Belas). Values represent the number of isolates for each enterococci species.

**Supplementary Table 4.** Comparative distribution of *Enterococcus* species across life cycle stages of *Heliconius erato phyllis* populations (PG1 and PG2).

|  |  | **Life stage ^2^** | | | | | | | | |  |
| --- | --- | --- | --- | --- | --- | --- | --- | --- | --- | --- | --- |
| **Population^1^** | **Species** | **PG** | **E** | **L1-C** | **L1-F** | **L2** | **L3** | **L4** | **L5** | **P** | **Total** |
| **PG1** | *E. casseliflavus* | 0 | 0 | 0 | 18 | 28 | 0 | 6 | 10 | 0 | **62** |
|  | *E. faecalis* | 17 | 0 | 0 | 0 | 0 | 0 | 48 | 50 | 0 | **115** |
|  | *E. mundtii* | 0 | 0 | 0 | 0 | 0 | 12 | 0 | 0 | 0 | **12** |
|  | *E. hirae* | 0 | 0 | 0 | 10 | 0 | 0 | 0 | 0 | 0 | **10** |
|  | **Subtotal** | **17** | **0** | **0** | **28** | **28** | **12** | **54** | **60** | **0** | **199** |
| **PG2** | *E. casseliflavus* | 0 | 0 | 0 | 10 | 10 | 10 | 10 | 0 | 45 | **85** |
|  | *E. faecalis* | 20 | 0 | 16 | 0 | 0 | 0 | 0 | 10 | 0 | **46** |
|  | *E. mundtii* | 0 | 0 | 0 | 0 | 0 | 0 | 0 | 10 | 0 | **10** |
|  | *E. hirae* | 0 | 10 | 0 | 0 | 0 | 0 | 0 | 0 | 0 | **10** |
|  | *E. faecium* | 0 | 1 | 0 | 0 | 0 | 0 | 0 | 0 | 0 | **1** |
|  | **Subtotal** | **20** | **11** | **16** | **10** | **10** | **10** | **10** | **20** | **45** | **152** |
| **Total** |  | **37** | **11** | **16** | **38** | **38** | **22** | **64** | **80** | **45** | **351** |

1. Population codes: PG1 (parental generation 1, Porto Alegre), PG2 (parental generation 2, Águas Belas). 2. Life stage abbreviations: PG (parental generation), E (eggs), L1-C (1st instar starved), L1-F (1st instar feeding), L2-L5 (larval instars), P (pupa - meconium).
